# Tenth Annual Meeting of the Illinois State Medical Society

**Published:** 1860-06

**Authors:** N. S. Davis

**Affiliations:** Permanent Secretary; Paris, Edgar Co., Ill.


### TENTH ANNUAL MEETING OF THE ILLINOIS STATE MEDICAL SOCIETY.

HELD IN PARIS, ILL., MAY 8TH AND 9TH, 1860.

FIRST DAY—MORNING SESSION.

The Society met in the Elliott Chapel, and was called to order at 10 o'clock, A. M., by Dr. H. W. Davis, one of the Vice-Presidents, the President being absent.

Dr. S. York, Chairman of the Committee of Arrangements, welcomed the members of the Society to the hospitalities of the profession and citizens of Paris, and reported the following delegates and permanent members as present:

DELEGATES.—J. A. W. Hostetter and Ira B. Curtis, of Decatur, from Macon, Co. Med. Soc.; R. H. Brown, of Mahomet, and John Swain, of Champaign, from Champaign Co. Med. Soc.; R. G. Laughlin, of Heyworth, T. D. Fisher, of LeRoy, from McLean Co. Med. Soc.; T. K. Edmiston, of Clinton, De Witt Co. Med. Soc.; J. H. Apperson, of Bourbon, Douglas Co. Med. Soc.; D. E. Foote, of Belvidere, Boone Co. Med. Soc.; D. W. Stormont, of Grandview, and L. L. Todd, of Paris, from Esculapian Med. Soc.; W. M. Chambers, of Charleston, Eberleon Med. Soc.; N. S. Davis, of Chicago, Medical Department of Lind University; J. W. Freer, of Chicago, Rush Medical College; E. L. Holmes, of Chicago, Charitable Eye and Ear Infirmary.

Permanent Members.—J. S. Whitmire, of Metamora, Woodford county; G. Beeman, and S. T. Trowbridge, of Decatur, Macon county; J. M Steele, of Grandview, and S. York, and John Tenbrook, of Paris, Edgar county; H. Rice Payne, of Marshall, Clark county; H. W. Davis, of Terre Haute, Ind., formerly of Paris.

The Secretary then read the roll of members, and distributed copies of Transactions containing the minutes of the last annual meeting.

The Society then took a recess of ten minutes to enable the delegates and members from each county represented, to report one of their number to act on a committee for nominating officers and filling standing committees for the ensuing year.

The Society having been called to order, the following were reported as members of the Nominating Committee:

W. M. Chambers, of Coles county; J. S. Whitmire, of Woodford county; T. K. Edmiston, of De Witt county; E. L. Holmes, of Cook county; John Swain, of Champaign county; J. P. Apperson, of Douglas county; R. G. Laughlin, of McLean county; D. E. Foote, of Boone county; Ira B. Curtis, of Macon county; H. Rice Payne, of Clark county; J. M. Steele, of Edgar county.

On motion of Dr. J. W. Freer, the Nominating Committee was requested to defer any action until 3 1-2 o'clock, P. M., as delegates from other counties were expected to arrive in the afternoon trains.

On motion of Dr. W. M. Chambers, the regular order of business was suspended for the purpose of electing permanent members.

L. D. Martin, of Shelbyville, Shelby county, proposed by W. M. Chambers.

Charles Johnson, of York, Clark county, proposed by H. R. Payne.

John H. Clark, of Decatur, Macon county, proposed by Ira B. Curtis.

Ezra A. Steele, of Chicago, Cook county, by N. S. Davis.

J. T. Pearman, of Elbridge, Edgar county, by D. W. Stormont.

A. H. Kimbrough, of Georgetown, Vermillion county, by Wm. M. Chambers.

D. O. McCord, of York, Clark county, by H. R. Payne.

Charles Gorham, of York, Clark county, by H. R. Payne.

On motion of Dr. W. M. Chambers, the above-named gentlemen were unanimously elected permanent members of the Society.

B. F. Swafford, M. D., of New Goshen, Ind., was nominated for a permanent member, when the question was raised whether it was proper to elect permanent members residing out of this State. The question was discussed by Drs. Edmiston, Whitmire, H. W. Davis, and N. S. Davis.

The following resolution was offered by N. S. Davis, and adopted:

*Resolved*, That the constitution of this Society does not contemplate the election of permanent members residing out of the State. But a permanent member of this Society, elected while residing in this State, does not lose his membership by merely moving out of it.

Dr. D. W. Stormont moved that a committee of three be appointed on unfinished business, which was seconded and adopted. The President appointed D. W. Stormont, T. K. Edmiston, and H. R. Payne, said committee.

Dr. B. F. Swafford, of New Goshen, Ind., and Dr. Hedges, of Clinton, Ind., were unanimously elected members by *invitation*.

On motion of Dr. W. M. Chambers, the delegates appointed at the last annual meeting to attend the meeting of the American Medical Association, were requested to report at 10 o'clock to-morrow morning, whether they can act as such delegates.

On motion, the Society adjourned to 2 o'clock, P. M.

AFTERNOON SESSION.

At 2 o'clock, P. M., the Society was called to order, Dr. S. T. Trowbridge, one of the Vice Presidents, in the chair.

The minutes of the previous session were read and approved.

The following named gentlemen were unanimously elected permanent members, viz:

J. W. Lawrence, M. D., of Carbondale, Jackson Co., proposed by S. York.

John W. Frizell, M. D., of Bloomfield, Edgar Co., by S. York.

J. D. Mitchell, M. D., of Darwin, Clark county, by H. R. Payne.

The following members were then added to the nominating committee:

Dr. L. D. Martin, of Shelby county,—A. H. Kimbrough, of Vermillion county,—John W. Lawrence, of Jackson county,—T. D. Fitch, of Henry county,—A. W. Heise, of Will county.

The reports of the standing committees being in order, a communication and report from Dr. C. Goodbrake, chairman of the Committee on Practical Medicine, was presented by the Secretary. After a considerable part of the report had been read by the Secretary, on motion of Dr. W. M. Chambers it was referred to the Committee of Publication, with discretion to publish so much as they deem proper.

During the reading of the report on Practical Medicine, the President, Dr. David Prince, of Jacksonville, arrived and took the chair.

The Committee of Arrangements also reported as present the following additional delegates and members.

Dr. T. D. Fitch, of Kewanee, from Henry Co. Med. Society.
" A. W. Heise, of Joliet, from Will county Med. Society.

Dr. A. R. Spears, of Kansas, from Esculapian Society.
" Daniel Brainard, of Chicago, from Rush Med. College.
" DeLaskie Miller, of Chicago, from City Hospital.
" David Prince, of Jacksonville, Permanent Member.

Dr. J. S. Whitmire, of Metamora, presented and read a report on the Treatment of Rheumatism, which was referred to the Committee on Publication.

The Committee on Drugs and Medicines being called, the Secretary read a letter from Dr. F. K. Bailey, of Joliet, chairman of the committee, apologizing for the failure to report, which letter was referred to the Nominating Committee.

The Treasurer, Dr. J. W. Freer, of Chicago, presented and read his annual report, as follows:

Illinois State Medical Society,

To J. W. Freer, *Treasurer*, Dr.

| | |
|---|---|
| To cash paid Wm. Cravens for printing transactions for 1859, | $158 00 |
| " " " Dr. J. W. Phillips, for Prize Essay, | 50 00 |
| " " " Dr. A. S. Hudson, Prize Essay, | 20 00 |
| " " " Postage, | 1 50 |
| | $229 50 |

Cr.

| | |
|---|---|
| By cash for annual assessment and initiation fees, | $190 00 |
| " " of Dr. N. S. Davis for Prize Fund, | 20 00 |
| " " Dr. Blaney former Treasurer Prize money, | 30 00 |
| " " Dr. J. M. Steele, Prize Essay on Opium, | 20 00 |
| | $260 00 |
| Balance in the Treasury, | $30 00 |

The report was accepted, and ordered to be printed in the proceedings of the Society.

The Committee to nominate officers for the ensuing year, presented the following report:

*For President,*

Wm. M. Chambers, M. D., of Charleston.

*For Vice Presidents.*

T. K. Edmiston, M. D., of Heyworth.

H. R. Payne, M. D., of Marshall.

*Treasurer.*

J. W. Freer, M. D., of Chicago.

Place for the next annual meeting, Jacksonville, Morgan county, Ill.

On motion, the report was accepted and adopted unanimously.

On motion, the Society adjourned until 8 o'clock, P. M., to hear the annual address.

EVENING SESSION.

At 8 o'clock, P. M., the Society, with a large audience of citizens, assembled in the Chapel, and were called to order by the President. Dr. N. S. Davis, of Chicago, then delivered the Annual Address. The subject was the "Mutual relations and consequent mutual duties of the medical profession and the community." Its delivery occupied three-quarters of an hour.

Dr. David Prince, of Jacksonville, the retiring President, then read an interesting valedictory address, on the legal relations and responsibilities of the physician and surgeon. It was listened to with marked attention and pleasure.

On motion, the Society adjourned to 9 o'clock, A. M.

SECOND DAY.—MORNING SESSION.

At 9½ o'clock, A. M., the Society was called to order by the President, Dr. D. Prince, who, after a few remarks, introduced the President elect, Dr. W. M. Chambers, to the chair. Dr. Chambers thanked the Society for the honor conferred upon him, and proceeded with the regular order of business. The Secretary read the minutes of the last meeting, which were corrected and approved.

The report of the Standing Committee on Obstetrics being called for, Dr. W. H. Byford, the chairman, was absent. He had prepared a report, however, which was presented by the Secretary, and an abstract of the same read, when on motion the report was referred to the Committee of Publication.

Dr. DeLaskie Miller of Chicago, from the Special Committee on the Hygiene and Sewerage of Cities, presented and read a report, which was accepted, and referred to the Committee of Publication.

On motion of Dr. S. York, the order of business was suspended, for the purpose of hearing from the delegates appointed at the last meeting of the Society, to attend the American Medical Association, and to fill such vacancies as may be found to exist.

Six of the delegates previously appointed announced themselves as unable to attend the coming meeting in New Haven, and their places were filled by the following: Z. H. Whitmire, of Metamora; John Tenbrook, of Paris; A. A. Dunn, of Cambridge; Charles Johnson, of York; A. H. Luce, of Bloomington; T. D. Fitch, of Kewanee.

Z. H. Whitmire, of Metamora, Ill., was proposed by W. H. Davis; A. A. Dunn, of Cambridge, Henry county, was proposed by T. D. Fitch, and both unanimously elected permanent members of this Society.

The hearing of reports from standing committees was again resumed.

Dr. D. Brainard, of Chicago, Chairman of the Committee on Surgery, presented his report, consisting of a paper written by himself; another by Dr. Powell, of Chicago, on Fractures; one by Dr. D. Prince, of Jacksonville, on Silver Sutures; and one by Dr. I. S. Whitmire, on the Bite of Serpents. He also exhibited to the Society several new surgical instruments, making extempore comments on their uses and utilty.

Dr. D. Prince moved that the report of Dr. Brainard be accepted and referred to the Committee of Publication, with instructions to print the same, which was adopted.

On motion, the Society adjourned.

SECOND DAY—AFTERNOON SESSION.

At 2 o'clock, P. M., the President, Dr. Chambers, called the Society to order. The Secretary, in behalf of the Committee of Publication, presented the following report, which was accepted, and its recommendations adopted, viz.:

*Report of Permanent Secretary in behalf of the Publishing Committee.*

As soon as practicable after the last Annual Meeting, the Transactions of the Society were sent to the printers for publication.

400 copies were printed, 150 of which have been distributed to members of the Society, the editors of Medical Journals, and the Secretaries of other State Medical Societies. 250 copies remain on hand for the use of the Society. The Transactions of all previous years remain as represented in the report of last year. The expense of printing the Transactions for 1859 was $158.00, as stated in the report of the Treasurer. Besides this, the Permanent Secretary has paid, in postage on copies of the Transactions distributed, and in letters in relation to the business of the Society, five dollars more, a bill for which accompanies this report.

From the fact that the printed copies of the Constitution and By-laws of the Society are nearly exhausted, your Committee would recommend that they, together with all amendments, be published with the transactions of the present meeting.

N. S. DAVIS,
Paris, Ill., May 9th, 1860. *Permanent Secretary.*

The Committee on Nominations then presented the following report, which was accepted and adopted :

That the annual assessment for 1860 be *two* dollars.

STANDING COMMITTEES.

*Committee of Arrangements.*—Drs. D. Prince, O. M. Long, A. McFarland, N. English, Henry Jones, of Jacksonville.

*Committee on Practical Medicine.*—Drs. S. T. Trowbridge, of Decatur, Macon county, John Swain, of Champaign, Champaign county, D. E. Foote, of Belvidere, Boone county.

*Committee on Drugs and Medicines.*—Drs. F. K. Bailey, of Joliet, Will county, R. G. Laughlin, of Heyworth, McLean county, H. R. Payne, of Marshall, Clark county.

*Committee on Obstetrics.*—Drs. T. D. Fitch, of Kewanee, Henry county, DeLaskie Miller, of Chicago, Cook county, J. B. Curtis, of Decatur, Macon county.

*Committee on Surgery.*—Drs. A. W. Heise, of Joliet, Will county, J. W. Freer, and E. Andrews, of Chicago, Ill.

SPECIAL COMMITTEES.

*Itinerant Practitioners.*—Drs. S. York, of Paris, Edgar co., J. P. Apperson, of Bourbon, Douglas county, John Wright, of Wapella, DeWitt county.

*Diseases of the Eye.*—Dr. E. L. Holmes, Chicago.

*Typhoid Fever.*—Drs. H. Noble, of Heyworth, McLean county; Hiram Nance, of Lafayette, Stark county; L. D. Martin, of Shelbyville, Shelby county.

*Stomatitis Materni.*—Dr. A. J. Crane, of Champaign City, Champaign county.

*Medical Electricity.*—Dr. J. A. W. Hostetter, of Decatur, Macon county.

*Assistant Secretary.*—Dr. O. M. Long, of Jacksonville.

Report from the Special Committee on Chlorosis being called for, Dr. E. W. Moore, chairman of the committee, was absent, but had sent a communication to the effect that he had been prevented from finishing his report by sickness, but would have it completed in a few days and leave it at the disposal of the Society. On motion, Dr. Moore was requested to finish his report, and forward it to the Committee of Publication.

Dr. A. Hard, of Aurora, chairman of the Committee on Veratrum Viride, had sent his report, which was read by the Secretary, and referred to the Committee of Publication.

Dr. E. L. Holmes, of Chicago, chairman of Special Committee on Diseases of the Eye, presented and read his report, which was accepted and referred to the Committee of Publication.

Dr. N. S. Davis presented and read an abstract of a paper on the food of infants when deprived of the milk of the mother; which was accepted and referred to the Committee of Publication.

Dr. N. S. Davis was requested to continue his researches as a Special Committee on the Changes in the Blood during continued Fevers.

Dr. D. W. Stormont, from the Committee on Prize Essays, reported that only one Essay had been received; and that came into the hands of a majority of the committee at so late a period that they have been unable to read it. The Committee therefore recommend the continuance of the offer for Prize Essays another year; and that the present essay remain as a competitor for it. The report of the committee was accepted and adopted.

Dr. DeLaskie Miller, of Chicago, was requested to continue as a Special Committee on the Hygiene of Cities.

Dr. H. W. Davis, of Terre Haute, Ind., was appointed a Special Committee on Serous Inflammation.

Dr. D. W. Stormont, Chairman of the Committee on Registration, made the following report, which was accepted and adopted:

"The Committee on the Registration Law, as instructed at the last meeting of the Society, would present the following petition for the signature of the officers, viz:

"*To the Hon. the General Assembly of the State of Illinois:*

"The undersigned, officers of the Illinois State Medical Society, in behalf of said Society, respectfully ask your Honorable Body to pass a law for the Registration of the Births, Marriages and Deaths in this State. Sanitary science demands it, and the interests of the people would be greatly advanced by it.

"Signed by order of the Society, adopted at the tenth annual meeting, held in Paris, Edgar county, May, 1860.

"The Committee have prepared a Law, which will be laid before the Legislative Committee having this matter in charge.

"We would request each local medical society to memorialize the Legislature on this subject. Will some member in each Society see that this matter is attended to?"

Respectfully submitted.

D. W. STORMONT,

*Chairman of Committee.*

Dr. J. S. Whitmire, of Metamora, offered the following resolution, which was adopted unanimously:

*Resolved,* That the thanks of this Society are due to the

Physicians of Paris in particular, and to the citizens generally, for the hospitable manner in which they have entertained its members during its present session. And that we will carry home with us the kindest regards for their future prosperity and welfare.

Dr. Brown offered the following resolution, which was adopted unanimously, and, together with that offered by Dr. Whitmire, was ordered to be printed in the local papers in Paris.

*Resolved*, That the thanks of the Illinois State Medical Society are hereby tendered to the Trustees and Members of the Methodist Episcopal Church, for the use Elliott Chapel during the present annual session of this Society.

Dr. D. W. Stormont, chairman of the Committee on Unfinished Business, reported that it would be proper to hear from the Committee on Legalizing Dissections, found on page 13 of Transactions for 1859; also from the Committee on Further Accommodations for the Insane; also from the committee to whom was referred the charges against Dr. White, of Salem; and to act on the resolutions offered by Dr. Washburn, and found on page 15 of the Transactions; and an amendment to the constitution found on page 18.

Dr. York, chairman of the Committee to Memorialize the Legislature in favor of Legalizing Dissections, reported verbally that there had been no session of the State Legislature since the last meeting of the Society, and the committee was continued another year.

Dr. Prince, chairman of the Committee on Further Accommodation for the Insane and Idiotic, reported verbally, that the work of providing further accommodations for the insane was already far advanced, but nothing was yet done in relation to the training of imbecile and idiotic children. The committee was continued, with instructions to memorialize the next Legislature in favor of suitable provision for the education of idiotic and imbecile children.

On motion of Dr. J. M. Steele, the resolutions found on page 15 of the Transactions for 1859, were taken from the table. On motion of Dr. S. York, their consideration was postponed until the commencement of the evening session.

Dr. W. M. Chambers, from the committee to which was referred the charges against Dr. Wm. White, reported that they had notified Dr. White of the charges preferred by Dr. Haller, and that he could be heard in defence at this meeting of the Society, or in any other way he might think best for the interests of all parties. The committee had received no reply or communication from him of any kind. After reading some of the evidence furnished by Dr. Haller in support of his charges, Dr. H. W. Davis moved that Dr. Wm. White, of Salem, be expelled from this Society, which was adopted by a unanimous vote.

The constitutional amendment found on page 18 of the Transactions for 1859, making the time of each annual meeting, as well as the place, depend on the vote of the Society, was taken up and adopted unanimously.

Dr. Prince moved that the time of holding the next annual meeting of the Society be on the first Tuesday of May or June, to be determined by notice of the Secretary, when it shall be known what time the meeting of the American Medical Association is to be held in 1861. Adopted.

On motion of Dr. R. Laughlin, Dr. T. K. Edmiston, of Clinton, was appointed a delegate to represent this Society in the next meeting of the Ohio State Medical Society, to be held at White Sulphur Springs.

On motion of Dr. Brown, Dr. John Swain, of Champaign, was appointed a delegate to represent this Society in the next annual meeting of the Kentucky State Medical Society.

On motion of Drs. T. D. Fitch, N. S. Davis and David Prince, were requested to furnish to the Committee of Publication copies of their addresses for publication in the Transactions of the Society.

On motion of Dr. T. D. Fitch, the Secretaries of Local Societies were requested to procure the publication of the address of Dr. N. S. Davis, in as many of the newspapers of their several localities, as possible.

Dr. J. H. Whitmire, of Metamora, was appointed a special committee to report on Erysipelas.

The following named members were duly nominated and elected delegates to attend the annual meeting of the American Medical Association for 1861:

Drs. A. W. Hostetter, of Decatur, ; J. S. Whitmire, of Metamora, Charles Johnson, of York; R. H. Brown, of Mahomet; T. D. Fitch, of Kewanee; J. D. Steele, of Grandview; Geo. Beman, of Decatur; S. York, of Paris; H. W. Davis, of Terre Haute, Ind; D. E. Foote, of Belvidere; D. Prince, of Jacksonville; W. D. Chambers, of Charleston; C. Goodbrake, of Clinton; H. R. Payne, of Marshall; C. N. Andrews, of Rockford.

On motion, the Society adjourned to 8 o'clock, P. M.

SECOND DAY.—EVENING SESSION.

At 8 o'clock, the Society was called to order by the President, Dr. W. M. Chambers.

Dr. O. Q. Herrick, of Kansas, permanent member, arrived and took his seat.

The president announced the following committee on Prize Essays for the ensuing year; Drs. H. A. Johnson, J. V. Z. Blaney, and E. L. Holmes, all of Chicago.

Dr. T. D. Fitch moved to take up the following resolutions, offered by Dr. Washburn at the last annual meeting of the Society.

*Whereas*, The American Medical Association is a national Association composed of delegates and members from all parts of the United States, meeting on terms of perfect equality:

Therefore, *Resolved*, That in the opinion of this Society, all the officers of the Association should be selected strictly with reference to *merit*, and without any regard to their place of residence.

*Resolved*, That the custom of selecting the President of the Association exclusively from the profession of the city in which the Annual Meeting is held, is not only derogatory to the general character of the organization, and calculated greatly to lessen the honor which should attach to that office, but past experience has shown that it leads directly to local divisions, jealousies, and injurious partisan strife.

*Resolved*, That the delegates from this Society to the Association, be instructed to use their influence to abrogate the custom alluded to in the preceeding resolution.

*Resolved*, That the Secretary be directed to furnish copies of the foregoing resolutions to other State and Local Medical Societies, and ask their attention to the same.

The motion was seconded by Dr. J. M. Steele, and after some remarks by Drs. Steele, Brainard, York, and Whitmire, the preamble and resolutions were adopted almost unanimously.*

The report of Itinerant Practitioners being called for, Dr. Curtis, one of the committee, made an amusing verbal report; after which, Dr. H. W. Davis, of Terre Haute, chairman of the committee, read a lengthy report in the form of a satirical poem, which was listened to with interest.

On motion of Dr. J. S. Whitmire, the thanks of the Society were tendered to Dr. Davis for his report, and a cane ordered to be presented as a token of their appreciation of its merits.

On motion of Dr. Brainard, the President was appointed to deliver the public annual address at the next meeting of the Society.

On motion of Dr. Brainard, the Society adjourned *sine die.*

N. S. DAVIS,

*Paris, Edgar Co., Ill.* *Permanent Secretary.*

*There being but five or six votes in its favor.—ED.

